# Modeling and Design of a Soft Capacitive Slip Sensor with Fluid Dielectric Interlayer

**DOI:** 10.3390/mi17030349

**Published:** 2026-03-12

**Authors:** Elia Landi, Tommaso Lisini Baldi, Michele Pallaoro, Federico Micheletti, Federico Carli, Ada Fort

**Affiliations:** Department of Information Engineering and Mathematical Sciences, University of Siena, 53100 Siena, Italy; elia.landi@unisi.it (E.L.); tommaso.lisini@unisi.it (T.L.B.); michele.pallaoro@unisi.it (M.P.); federico.micheletti@unisi.it (F.M.); federico.carli@unisi.it (F.C.)

**Keywords:** slip sensor, capacitive sensor, displacement sensor, soft robotics, robotic sensors, grasp monitoring

## Abstract

This paper presents the design, modeling, and experimental validation of a capacitive tactile sensor specifically conceived to sense shear-driven contact dynamics in robotic manipulation. The proposed device is a layered flexible capacitive structure, in which controlled tangential interactions are induced. The electrode design maximizes sensitivity to shear motion and promotes an isotropic response with respect to slip direction, thereby addressing two key limitations that affect the majority of existing slip-sensing technologies. An analytical model was developed to describe the essential relationship between shear-induced displacements and the electrical response, providing insight into the design parameters and supporting the selection of geometry and materials. To test the sensor in real conditions, a dedicated capacitive readout circuit based on high-frequency excitation and synchronous demodulation was developed to robustly acquire capacitance variations while rejecting static offsets and parasitic effects. Several formulations for the interposed dielectric layer material were investigated, including viscous fluids and composite mixtures with high-permittivity nanoparticles, with the aim of improving electrical sensitivity while preserving mechanical stability. Experimental results obtained under controlled loading and sliding conditions demonstrate that the sensor is highly sensitive to changes in contact state and tangential interaction dynamics. The sensor responded consistently to both load-induced shear and slip-related phenomena, enabling the reliable monitoring of contact dynamics rather than binary slip detection. A proof-of-concept integration into a robotic finger confirms the suitability of the proposed approach for grasp monitoring.

## 1. Introduction

In robotic manipulation, tactile sensing plays a central role in enabling stable grasping and adaptive interaction with objects by providing information about contact conditions, tangential interactions, and surface dynamics. From a physical point of view, grasp stability is driven not only by the magnitude of the normal force but also by the evolution of tangential stresses at the contact interface, which are tightly coupled to frictional behavior and surface properties. Classical contact and manipulation models have demonstrated that variations in tangential force and relative micro-motion at the interface precede gross slip and strongly affect grasp controllability and force distribution [1,2,3]. Consequently, variations in shear-related contact dynamics are widely recognized as early indicators of forthcoming slip, motivating extensive research on tactile sensing strategies for contact-aware and slip-aware control.

Existing literature on contact-based control (e.g., force, impedance, and tactile feedback) often takes for granted the availability of sensing modalities that can accurately resolve contact dynamics between the gripper and the object [1,2,3,4]. In practice, however, this assumption is only partially satisfied. Current control frameworks frequently replace direct sensing with approximations: simplified models, idealized friction assumptions, or indirect signals like motor currents and kinematic measurements [5,6]. These strategies can provide useful information under controlled conditions but often fail to generalize across different objects, materials, and complex geometries.

Despite decades of research, achieving reliable and universally applicable slip sensing remains an open challenge.

As highlighted in multiple surveys and review papers on tactile sensing and slip detection [7,8,9,10], slip-related phenomena emerge from a complex interplay of local frictional conditions, surface texture, material compliance, contact geometry, and transient dynamic effects. Incipient slip is characterized by localized micro-slip, partial detachment, and stick–slip transitions that occur well before macroscopic relative motion becomes observable. These processes are inherently dynamic and highly dependent on both the contacting materials and the mechanical compliance of the sensing interface. As a result, no existing sensing solutions can provide robustness, low cost, compactness, ease of integration, and consistent performance at the same time across a wide range of objects and operating conditions.

Vision-based and visuotactile sensors have recently gained considerable attention due to their ability to capture rich spatial information about contact deformation, shear, and slip patterns. Systems based on optical imaging of elastomer deformation, such as GelSight and related architectures, can directly observe surface strain fields and relative motion at the contact interface, enabling the detailed analysis of frictional behavior and incipient slip [11,12,13,14]. However, these approaches typically require bulky optical hardware, high computational resources, and controlled illumination conditions. Moreover, their form factor, bandwidth, and power consumption often limit their applicability to compact, fast, or wearable robotic grippers, as discussed in recent reviews on visuotactile sensing [15].

Alternative approaches based on vibration sensing exploit the micro-vibrations generated during stick–slip transitions and frictional instabilities. Tactile sensors embedding MEMS accelerometers or biomimetic vibration-sensitive structures can achieve high temporal resolution and have demonstrated effectiveness in detecting slip-related events [16,17,18]. Nevertheless, vibration-based methods are intrinsically sensitive to external shocks, structural resonances, and global vibrations of the robotic system. They also require strong and repeatable mechanical coupling between the sensor and the object, which limits their robustness in unstructured environments and complicates their integration into soft or highly compliant robotic fingers.

Other tactile sensing technologies (e.g., resistive, piezoresistive, piezoelectric, capacitive, and magnetic tactile arrays) can provide distributed measurements of pressure or deformation and have been extensively explored for robotic manipulation [7,8,9,10]. However, many of these solutions suffer from cross-axis coupling between normal and tangential loads, anisotropic responses, difficulty in isolating shear-related information, and long-term stability issues due to drift, hysteresis, or material aging. Consequently, although slip detection is frequently cited as a prerequisite for robust grasp control, sensing technologies capable of providing simple, compact, and reliable measurements of tangential contact dynamics, rather than binary slip events, remain limited.

Within this context, we present a tactile sensing from an instrumentation-driven perspective and focuses on the characterization of shear-related contact dynamics rather than on binary slip detection. In more detail, this paper investigates the development of a capacitive tactile sensing approach that exploits a fluid dielectric capacitance to capture variations in contact conditions during object interaction.

The proposed sensor is based on a layered capacitive configuration in which a thin viscoelastic dielectric medium is interposed between two flexible electrodes. Under tangential loading, relative shear-induced motion within this interlayer produces controlled variations in the effective overlap area of the electrodes, resulting in measurable changes in capacitance.

A key feature of the proposed architecture is the adoption of stacked concentric electrodes specifically conceived for integration on the outer surface of a robotic finger. The sensing electrodes are covered, on the side exposed to the external environment, by a continuous metallic layer that acts as an electrostatic shield. This external electrode is permanently connected to the voltage reference and is mechanically bonded to the sensing structure through a relatively stiff solid dielectric layer, ensuring an almost fixed relative position with respect to the sensor body.

The presence of the fluid dielectric enables the electrodes to slide smoothly with respect to each other during slip, converting tangential micro-displacements into variations of the overlap area and, consequently, of the measured capacitance.

One of the two ring electrodes is electrically shorted to the external shield, so that the active sensing capacitance is formed between the moving ring electrode and the reference-connected layer. This configuration preserves effective electrostatic shielding while maximizing sensitivity to shear-induced relative motion.

In addition, the rigid dielectric layer between the shield and the underlying electrode allows the capacitance between these two layers to be measured independently. Since this capacitance is primarily affected by normal compression, it can be used as an indirect proxy of the normal load applied to the sensor.

By multiplexing the measurement chain and selectively switching the electrode connections, the same front-end electronics can be time-shared to acquire both the shear-sensitive capacitive channel and the normal-load-related capacitive channel without increasing the hardware complexity. The concentric-ring electrode geometry maximizes sensitivity to tangential slip and promotes an approximately isotropic response with respect to slip direction, while the stacked, fluid-mediated, and shielded configuration improves robustness against parasitic coupling and environmental disturbances.

The stacked structure is encapsulated in silicone rubber to provide mechanical stability. It is important to note that the proposed sensing principle responds to both load-induced shear and slip-related contact dynamics; therefore, the sensor output should be interpreted as a contact dynamics indicator rather than a standalone slip detector. By prioritizing structural simplicity, compactness, and ease of integration, the proposed design aims to explore the feasibility of shear-induced capacitive sensing as a low-cost tactile modality.

Unlike vibration-based, optical, or feature-driven slip detection approaches, the sensing principle proposed in this work directly couples tangential displacement to a measurable electrical quantity by construction. Rather than inferring slip from secondary effects such as friction-induced vibrations, image-based deformation patterns, or resistive changes requiring advanced signal processing, the proposed architecture converts shear-induced relative motion into a controlled variation of electrode overlap area. This results in capacitance changes in the picofarad range, enabling a simpler analog front-end and a more direct physical interpretation of the measured signal. The objective is therefore not to maximize spatial resolution or high-frequency content, but to provide a structurally simple, compact, and computationally lightweight sensing modality for monitoring contact dynamics in robotic grasping.

The rest of the paper is organized as follows. Section 2 introduces a simplified analytical model of the sensing mechanism, highlighting the relationship between shear-induced deformation and the capacitive response. Section 3 describes the sensor design, fabrication process, and the dedicated capacitive readout electronics. Section 4 presents the experimental characterization of the sensor under controlled loading and sliding conditions, including durability tests and an evaluation of the influence of normal load. Section 5 reports proof-of-concept experiments performed on a robotic finger during grasping and slip scenarios. Finally, Section 6 discusses the main results and outlines directions for future work.

## 2. Sensor Operation and Design

Conventional capacitive tactile sensors typically rely on the compression of a solid dielectric layer between two conductive plates. While effective for pressure estimation, this configuration provides limited sensitivity to lateral displacement and is not ideal for measuring slippage, particularly in soft robotic systems where the contact surface is compliant and continuously deformable.

To overcome these limitations, in this work, a flexible capacitive sensor was developed based on a viscous dielectric layer instead of a solid one. The two electrodes were printed on a flexible substrate and separated by a thin encapsulated fluidic layer that responds to tangential motion by changing the overlapped area of the plates between the plates.

In fact, the device was based on two flexible electrodes separated by a thin encapsulated viscous layer acting as the dielectric and mechanical interface. When tangential forces occur at the fingertip–object interface, the two electrodes slide slightly with respect to each other, while the viscous medium allows relative motion and provides damping. This sliding causes a variation in the effective overlap area between the electrodes, which directly leads to a measurable change in capacitance.

The electrodes were patterned with circular symmetry so that a tangential displacement in any direction produces an equivalent change in the overlapping area. This configuration yields a direction-independent response to slip, an essential feature for integration in a soft robotic fingertip where the direction of contact forces cannot be predicted.

### 2.1. Sensor Model

The sensor structure consists of two flexible electrodes that are separated by a gap d filled with a viscous dielectric (relative permittivity $\varepsilon_{r}$; absolute $\varepsilon =\varepsilon_{0}\varepsilon_{r}$). Tangential slip produces a planar relative displacement vector r(t) (magnitude r(t) = ∥r(t)∥), changing the overlap area A(R, r), whereas motion along the direction perpendicular to r(t), n, due to grasping pressure, causes the plate distance, *d*, to change.

The capacitance of the sensor can be written:

(1) $$C=\frac{\varepsilon \;A\left(R,\;r\right)}{d}+C_{par}=C_{0}+C_{par}$$

with $C_{par}$ being the (constant) parasitic/shield capacitance, and R is the radius of the plates supposed circular.

Under the action of external forces, the capacitance changes due to the variations of its geometry, and for small geometrical changes, a linearized model can be used in which:

(2) $$dC=\frac{\partial C}{\partial A\left(R,r\right)}\;dA+\frac{\partial C}{\partial d}\delta =\frac{\epsilon }{d}\;dA-\frac{C_{0}}{d}\delta =\frac{\epsilon }{d}\frac{dA}{dr}\;r-\frac{C_{0}}{d}\delta$$

where δ is a small displacement along n.

For circular overlapping plates with radius R, using a cylindrical reference system and considering a displacement of r in any direction, or 0≤r≤2R in the overlap area, $A_{\circ }\left(R,\;r\right)$ becomes the standard circle–circle intersection:

(3) $$A_{\circ }(R,\;r)=2R^{2}{cos}^{-1}\;(\frac{r}{2R})-\frac{r}{2}\sqrt{4R^{2}-r^{2}}.$$

With small displacements (r≪R), Equation (2) can be approximated by the following equation:

(4) $$A_{\circ }\left(R,\;r\right)\approx \pi R^{2}-2rR-\;rR\Rightarrow \frac{dA_{\circ }}{dr}\approx -2R.$$

So, Equation (2) becomes:

(5) $$dC=-\frac{\epsilon }{d}2Rr-\frac{C_{0}}{d}\delta$$

Upon the application of external forces during grasping, the capacitance changes according to the following equation:

(6) $$C\left(r,\delta \right)=C_{par}+C_{0}\left(1-\frac{\delta }{d}\right)-2\frac{\epsilon }{d}Rr$$

The capacitance depends both on r as desired, and on δ. Moreover $C_{0}$ behaves as a large offset.

**Figure 1:**
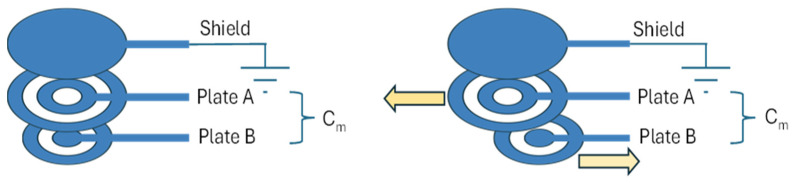
Schematic structure of the flexible capacitive slip sensor with the viscous dielectric layer. Arrows show the sliding directions of the substrates.

To counteract the unwanted sensitivity towards the pressure effect (δ), the geometry shown in Figure 1 was used, in which the two plates consist of two concentric rings with external and internal radii $R_{{out}_{j,k}}$, $R_{{in}_{j,k}},$ respectively, with k = 1, 2 and j = 1, 2 (k indicates the ring, j the plate). The design choices were such that:

(7) $$R_{in_{1,1}}<R_{in_{1,2}}<R_{{out}_{1,1}}<R_{in_{2.1}}<R_{{out}_{1,2}}<R_{in_{2,2}}<R_{{out}_{2,1}}<R_{{out}_{2,2}}$$

With $(R_{{out}_{j,k}}-R_{{in}_{j,k}})=h,\;for\;each\;j,k.$ Each ring can be written as the difference between two circles (an electrode and a hole), so:

(8) $$A_{{ring}_{j,k}}\left(R_{{out}_{j,k}},R_{{in}_{j,k}},0\right)=A_{o}\left(R_{{out}_{j,k}},0\right)-A_{o}\left(R_{{in}_{j,k}},0\right)$$

And the overlapping areas are:

(9) $$A_{over}\left(r=0\right)={A_{ring}}_{1}\left(\left(R_{{out}_{1,1}},R_{in_{1,2}}\right)\;,\left(R_{{out}_{1,1}},R_{in_{1,2}}\right),\;0\right)\;+{A_{ring}}_{2}\left(\left(R_{{out}_{1,2}},R_{in_{2,1}}\right)\;,\left(R_{{out}_{1,2}},R_{in_{2,1}}\right)\;,0\right)+\;{A_{ring}}_{3}\left(\left(R_{{out}_{2,1}},R_{in_{2,2}}\right)\;,\left(R_{{out}_{2,1}},R_{in_{2,2}}\right)\;,0\right)$$

where ${A_{ring}}_{1}$ is considered if $R_{in_{1,2}}<R_{{out}_{1,1}}$, ${A_{ring}}_{2}$ if $R_{in_{2,1}}<R_{{out}_{1,2}}$ and ${A_{ring}}_{3}$ if $R_{in_{2,2}}<R_{{out}_{2,1}}$.

The radii are chosen as follows:

(10) $$R_{in_{1,1}}=0\;\mathrm{cm}<R_{{out}_{1,1}}=R_{in_{1,2}}<R_{{out}_{1,2}}=R_{in_{2,1}}<R_{{out}_{2,1}}=R_{in_{2,2}}<R_{{out}_{2,2}}$$

Therefore, the overlapping area is equal to 0 if r = 0, and the sensor capacitance is $C=C_{par}$ since $C_{0}=0\;pF,$ and will not change upon the application of a normal force causing a displacement δ.

The sensor capacitance changes when r ≠ 0. Considering again small displacements (and r < $R_{{out}_{j,k}}-R_{{in}_{j,k}})$, the sensor plates non overlapping areas can be described by two circles with the same center when r = 0 and radii $R_{{out}_{1,1}}$ and $R_{{out}_{2,1}}$ (same geometry treated before), and the variations of the non-overlapping area ${dA}_{No1}\left(r\right)\approx -2R_{{out}_{1,1}}r$ and ${dA}_{No2}\left(r\right)\approx -2R_{{out}_{2,1}}r$. Therefore, the overlapping area will vary as: ${dA}_{o}\left(r\right)=-{dA}_{No1}\left(r\right)-{dA}_{No2}\left(r\right)$, i.e., ${dA}_{o}\left(r\right)\approx 2R_{{out}_{1,1}}r+2R_{{out}_{2,1}}r.$ So finally:

(11) $$C\left(r\right)=C_{par}+2\frac{\epsilon }{d}(R_{{out}_{1,1}}+R_{{out}_{2,1}})r$$

Equation (11) implies that for small displacements, before the full engagement of all ring pairs, the sensor behaves approximately as if the overlapping area increased linearly with the slip amplitude, with a proportionality constant given by the sum of the active outer radii of the first engaged rings, and the sensor sensitivity can be simply written as follows:

(12) $$s=\;\frac{dC}{dr}{|}_{r\to 0}\approx 2\frac{\varepsilon }{d}(R_{{out}_{1,1}}+R_{{out}_{2,1}})$$

The proposed electrode design compensates for the sensor response to displacement in the normal direction, nevertheless this displacement has an effect on the sensitivity, which depends inversely on the plate distance ($\frac{ds}{dd}=-\frac{s}{d}$): the smaller the plate distance, the larger the sensitivity. As this was a slip sensor designed for enhancing grasping in soft robotics, the normal force exerted on the sensor is always of positive sign, i.e., tends to reduce the plate distance. Moreover, the task of the sensor is to detect slip promptly, so the sensor must be designed to provide a value of s sufficiently large for δ=0. The enhanced sensitivity under deformation due to grasping force will only cause a prompter response of the control system.

Note that in the sensor model, the fringe capacitance is not accounted for. On the contrary it plays a role in the sensor behavior, which can be described by an additive term, $C_{fringe}(r,\;\delta )$, in Equation (1), such that:

(13) $$C\left(r,\delta \right)=C_{par}+2\frac{\epsilon }{d}(R_{{out}_{1,1}}+R_{{out}_{2,1}})r+C_{fringe}(r,\;\delta ).$$

The added term depends in a complex way on the plate distance and geometry, when r = 0 mm can be written as:

$$C_{fringe}\left(0,\delta \right)=2R_{{out}_{1,1}}\epsilon \log\left(1+\frac{R_{{out}_{1,2}}-R_{{out}_{1,1}}}{d-\delta }\right)+2R_{{out}_{2,1}}\epsilon \log\left(1+\frac{R_{{out}_{2,2}}-R_{{out}_{2,1}}}{d-\delta }\right)$$

Its contribution is maximum when r = 0, causing a difference between the linear model and the real behavior.

The electrodes of the proposed sensor are deposited on polymer substrates, resulting in a multilayer stack composed of two solid thin dielectric layers and the fluidic layer. This configuration can be modeled as three capacitors in series. Therefore, the model derived before remains valid but uses an equivalent dielectric constant, calculated as follows:

(14) $$\epsilon_{eq}=\epsilon_{solid}\epsilon_{fluid}\frac{d_{solid}+d_{fluid}}{\epsilon_{solid}d_{fluid}+\epsilon_{fluid}d_{ksoolid}}$$

### 2.2. Dynamic Model and Design Choices

To describe the sensor dynamic, a lumped parameter model can be used; this allows for predicting its approximate behavior and evaluating the suitability for the application, in which slip must be detected within a time short enough to manage the control action.

In this configuration, the viscous layer produces Couette shear drag and the encapsulation contributes an effective tangential stiffness k (from silicone, adhesive, cable routing, etc.). Let m be the effective moving mass (usually negligible).

Let n be the contact normal (pointing into the sensor) and t is the tangent along the slip direction.

Equation of motion along the slip direction (1D for clarity; extend to vector r component-wise):

(15) $$m\;\ddot{r}+b\;\dot{r}+k\;r=F_{t},b=\eta \;\frac{A_{shear}}{d},$$

where $F_{t}$ is the total force component in the slip direction, η is the dynamic viscosity (Pa·s), and $A_{shear}$ the wetted area. In most devices, m is tiny, therefore, an overdamped first order dynamic is:

(16) $$b\dot{\;r}+k\;r=F_{t}$$

where $F_{t}=F_{g,t}+F_{ext}-\mu_{k}N\;sgn(\dot{r})$, with $F_{g,t}=m_{object}g\;cos\theta$ being the effect of the weight of the grasped object (g gravity acceleration, $m_{object}$ is the mass of the object, θ is the angle between the normal to the sensor and gravity), N = $-mgcos\theta +N_{actuator}$ is the grasping force in the direction n, and $\mu_{k}$ is the dynamic friction coefficient.

The time constant of this simple first-order dynamic description is $\tau =\frac{b}{k}=\frac{\eta A_{shear}}{k\;d},$ which determines how rapidly the sensor responds to tangential motion.

This increases with the dynamic viscosity η of the fluid and with the sheared area $A_{shear}$, while it decreases with the effective tangential stiffness k of the encapsulating silicone layer and with the gap d. Increasing viscosity therefore enhances damping and stabilizes the mechanical response, but at the cost of a slower dynamic behavior. Conversely, low-viscosity fluids yield a faster response but allow high-frequency disturbances and micro-vibrations to propagate to the sensing element.

This trade-off is particularly relevant for slip detection. Quasi-static variations of capacitance are strongly influenced by the object geometry, material compliance, and equilibrium grasping conditions, and are therefore poorly suited for absolute slip estimation. On the other hand, slip events manifest as rapid local variations in tangential displacement, consisting also of intermittent stick–slip, detachment, and re-adhesion processes at the contact interface. These phenomena typically populate a frequency range spanning from a few Hz (incipient slip and micro-creep) up to several hundreds of Hz (classical stick–slip and surface-induced micro-vibrations), depending on the contacted material (metal, plastic, rubber, or fabric) and on the compliance of the silicone encapsulation.

As a consequence, the sensor should be designed so that its mechanical bandwidth lies above the frequency range of interest for slip dynamics (10–100 Hz) while remaining below the dominant vibration spectrum of the robotic structure, in order to provide intrinsic mechanical filtering.

Assuming a shear area of $A=3\;{\mathrm{cm}}^{2}$, a gap $d=d_{kapton}+d_{fluid}=300\;\mu m$, and a tangential stiffness $k=1\times 10^{5}\;N/m$, the performance obtained using different fluids are summarized below.

The increase in area amplifies the damping effect of the viscous layer proportionally, thus extending the time constant by about one order of magnitude compared with smaller prototypes. With low-viscosity fluids such as alcohol or light silicone oils, the mechanical bandwidth remains in the hundreds to thousands of hertz, which is too high to attenuate the micro-vibrations typical of soft robotic structures. Highly viscous greases or petrolatum-based blends, on the other hand, reduce the bandwidth to below 1 Hz, resulting in an overly sluggish response. A medium-viscosity range (η ≈ 0.1–1 Pa·s, e.g., silicone oils from 100 cSt to 1000 cSt) ensures balanced behavior: the sensor reacts fast enough to detect incipient slip (10–100 Hz range) while naturally filtering high-frequency disturbances and contact chatter.

Note that the proposed dynamic model is intentionally simplified: it does not capture high-viscosity effects such as adhesion, shear-locking, or other non-Newtonian behaviors that may appear with very thick fluids or very thin layers. These omissions, however, do not compromise the intended use of the model: it serves as a practical guideline to orient design choices and provides a concise description of the system dynamics, which is sufficient for detecting slippage in robotic gripping applications.

## 3. Sensor Fabrication

**Figure 2:**
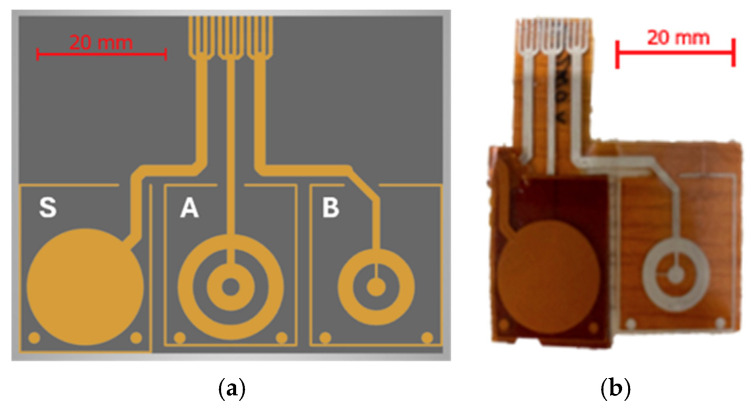
(**a**) Sensor printing layout. (**b**) Printed sensor plates.

A 75 μm thick polyimide (PI) sheet was used as a flexible substrate. The electrodes were printed on the substrate according to the layout shown in Figure 2a.

Printing was carried out using both a Dimatix inkjet piezoelectric printer (Fujifilm, Tokyo, Japan) and a Voltera Nova printer (Voltera, Waterloo, ON, Canada), following the manufacturers’ instructions with minor adjustments. In the long-term, the Voltera printer was preferred due to its comparable printing precision and superior ease of use.

Given the need to withstand significant mechanical stress in specific regions, a stretchable silver ink (ACI SS1109, Goleta, CA, USA) was employed. After printing, curing was performed in accordance with the ink manufacturer’s specifications.

The sensor is composed of three parts, as shown in Figure 1 and Figure 2a:

- A ground plate (S), used as an electrical shield and placed on top of the stacked sensor structure;
- The upper capacitive plate (A);
- The lower capacitive plate (B).

The sensing structure is obtained by cutting and folding the printed PI substrate as shown in Figure 2b. A solid adhesive dielectric layer is used to rigidly bond the shield (plate S) to electrode A, while a fluid dielectric layer is placed between electrodes A and B. This results in a stacked capacitive configuration in which two electrodes (A and S) are mechanically constrained by a relatively rigid dielectric layer. In this arrangement, the outer electrode is primarily exploited as a grounded electrostatic shield, improving immunity to parasitic coupling and environmental interference.

The plate design of the shear-sensing capacitor is based on the non-overlapping concentric rings structure described in the previous section (plates A and B in Figure 2a). For the prototype, the radii were chosen as follows:

$${R_{in}}_{1,1}=0\;\mathrm{cm},\;{R_{out}}_{1,1}={R_{in}}_{1,2}=1.25\;\mathrm{mm},\;{R_{out}}_{1,2}={R_{in}}_{2,1}=3.25\;\mathrm{mm},\;{R_{out}}_{2,1}={R_{in}}_{2,2}=5.2\;\mathrm{mm},\;{R_{out}}_{2,2}=7.25\;\mathrm{mm}$$

The sensor was assembled with the following procedure. Each part was cut along its perimeter and unnecessary portions were removed. Once cut to its final shape, the ground plate (with the conductive side facing up) was covered with two additional 75 μm thick PI layers, which were firmly stacked on top of it using a bi-adhesive tape. The second PI layer was then covered with an additional bi-adhesive tape to bond it face-to-face with the upper capacitive plate of the sensor. Alignment dots were used to accurately center each plate with respect to the subsequent one during assembly.

At this stage, the sensor was turned face down, and a few milligrams of viscous dielectric fluid were uniformly applied to the back of the upper capacitive plate. The lower capacitive plate was then folded and bonded back-to-back with the upper plate.

**Figure 3:**
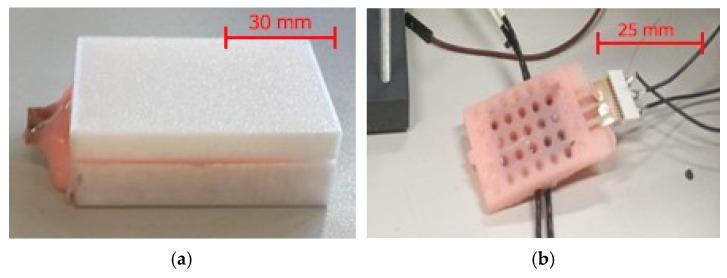
(**a**) 3D-printed mold and (**b**) sensor prototype.

**Figure 4:**
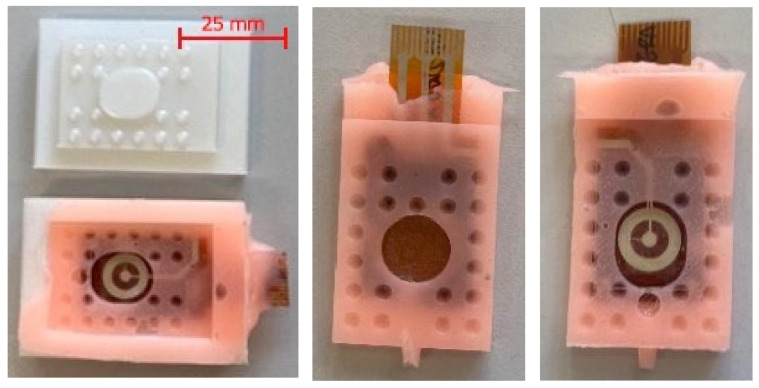
Mold and sensor prototype. Special design for characterization purposes with a window making the plates accessible. Leftmost: before removing extra silicone, center: top view, rightmost: back view.

The assembled structure was subsequently placed into an ad hoc designed mold, as shown in Figure 3a, which enabled accurate positioning of the two plates, including precise control of their spacing (nominal value of 300 µm). The mold also allowed for controlled deposition of the fluid dielectric between the plates and final encapsulation of the entire structure in liquid silicone (RPRO10) (Rechimica, Barberino Tavarnelle, Italy), which was cast and left to cure until solidification. Figure 4 shows the final prototype.

Due to the stacked configuration, the capacitance between the two fixed electrodes (S and A) is predominantly affected by normal compression rather than by tangential displacements. This property can be exploited to obtain an indirect estimate of the normal load $F_{n}$ by selectively measuring the corresponding capacitance. By multiplexing the readout chain and switching the electrode connections, the same front-end electronics can be time-shared to acquire both a normal-load-related capacitive channel (Capacitance A–S) and a shear/contact-dynamics-related channel (capacitance A–B). This dual-use configuration enables compact sensing architecture while providing additional information to support the interpretation and normalization of shear-induced responses.

Electro-mechanical coupling effects between the layered capacitive elements and the external environment are minimized by design. The full-area electrode (plate S) acts as a grounded electrostatic shield under normal operating conditions, significantly reducing parasitic capacitive coupling with external objects and the robotic structure. Moreover, this shield is rigidly bonded to the adjacent plate through a stiff dielectric layer, so that relative motion is confined to the intended viscous interlayer (A–B). For optimal installation, the shielded side is oriented toward the external contact interface, while the opposite electrode faces the mechanical support. This configuration limits cross-coupling effects and preserves consistency between the predicted and measured responses.

## 4. Experimental Setup

### 4.1. Test Bench for Sensor Characterization

To characterize the capacitive slip sensor and to validate the analytical model, a dedicated test bench was developed to generate precise and repeatable relative sliding between the two electrodes A and B while simultaneously acquiring impedance data. This setup enables a direct correlation between the imposed tangential displacement and the measured capacitance variation, which is essential for validating both the analytical model and the dynamic behavior of the device.

The relative motion between the plates is produced by a high-resolution linear piezoelectric actuator (PiezoLegs LL06, Acuvii, Uppsala, Sweden), chosen for its ability to generate micrometric steps with a nominal 5 μm stroke resolution and an integrated encoder providing 0.08 μm positional resolution while delivering up to 6.5 N of actuation force. The actuator drives the carriage of a linear recirculating-ball guide, ensuring unidirectional motion with minimal friction and negligible mechanical backlash. This configuration minimizes any unintended slipping between the two plates during the tests. The sensor is mounted within a custom structure manufactured through additive 3D printing techniques, designed to maintain accurate electrode alignment and to transfer the imposed displacement faithfully to the active sensing layers. Mechanical coupling between the actuator and the sensor is achieved through a surface featuring a pyramidal lattice, which conforms elastically to the silicone outer layer and prevents micro-slippage, and by exploiting special prototypes (shown in Figure 4), exposing the PI surfaces that can be rigidly attached to the sliding structures. This aspect is particularly important given that the characterization relies on a precise positional reference between the electrodes.

The test bench accommodates both the designed prototypes equipped with the characterization window, allowing their rigid attachment to the actuator and the fully encapsulated silicone sensors to also assess the influence of the external soft layer.

**Figure 5:**
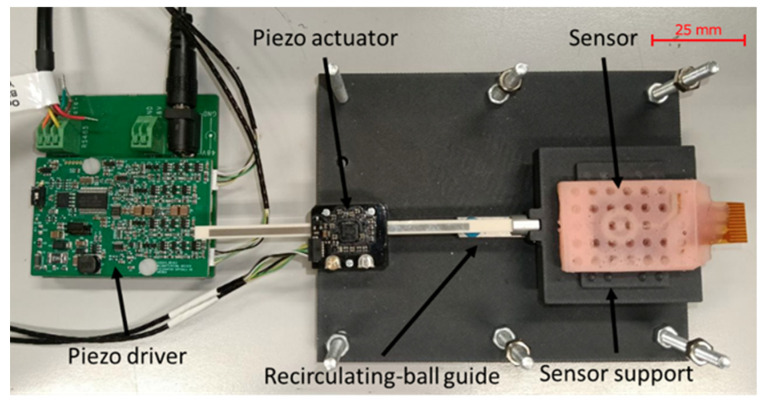
Experimental setup. The system consisted of a Piezo driver (Piezolegs, Acuvi, Uppsala, Sweden) controlling a Piezo actuator mounted on a recirculating-ball guide for linear motion. Note that the top cover, which holds the sensor, is not mounted.

The entire test bench (Figure 5) is automated through a custom virtual instrument (LabVIEW 20.0, Austin, TX, USA) that controls the actuator, acquires the encoder readings, and interfaces with the impedance analyzer. During the characterization procedure, the actuator moves in a sequence of known micrometric steps; after each step, the system ensures mechanical stabilization and then initiates electrical acquisition. This approach enables the accurate reconstruction of the capacitance–displacement curve and allows for the evaluation of sensitivity, linearity, repeatability, and potential hysteresis.

Electrical measurements were performed using both a Wayne Kerr 6500B (Wayne Kerr Electronics, West Sussex, UK) impedance analyzer and tailored front-end electronics developed for capacitance measurements.

### 4.2. Front-End Electronics

A dedicated electronic front-end was developed to acquire and condition the signal generated by the sensor and to enable dynamic measurements of capacitance variations induced exclusively by mechanical excitations. The adopted architecture was based on high-frequency excitation and synchronous demodulation, which allows small capacitance changes to be converted into a low-frequency voltage signal with high sensitivity and robustness against parasitic effects.

The sensing capacitance *C*_*s*_ is excited by a sinusoidal voltage, $v_{exc}\left(t\right)=V_{exc}sin(\omega_{0}t)$, where $\omega =2\pi f_{0}$ and $f_{0}$ is a fixed high frequency, typically 10 MHz, and connected in series with a shunt resistor *R*_*s*_. The value of *R*_*s*_ was carefully selected to minimize the influence of parasitic capacitances in parallel with the resistor at the operating frequency. The value was selected in order to have negligible impedance compared to the sensor impedance, ensuring that the measured signal is dominated by the current associated with the sensor capacitance.

Under this assumption (*R_s_* ≈ 0 Ω), the voltage across *R*_*s*_ is:

(17) $$v_{R_{s}}\left(t\right)=R_{s}i\left(t\right)=\omega_{0}R_{s}C_{s}V_{exc}cos(\omega_{0}t)$$

Assuming that the sensor capacitance can be expressed as $C_{s}=C_{0}+\Delta C(t)$, where $C_{0}$ is the nominal capacitance and $\Delta C\left(t\right)\ll C_{0}$ is the time-varying capacitance change induced by mechanical slip, Equation (16) becomes:

(18) $$v_{R_{s}}\left(t\right)=\omega_{0}R_{s}V_{exc}\left[C_{0}+\Delta C\left(t\right)\right]cos(\omega_{0}t)$$

The voltage across *R*_*s*_ is amplified by a first high-frequency gain stage implemented using a wideband current-feedback operational amplifier (OPA695, Texas Instruments, Dallas, TX, USA), having a bandwidth up to 100 MHz, which guarantees adequate gain and phase margin at the excitation frequency and preserves signal integrity prior to demodulation.

The amplified signal is then applied to a mixer and multiplied by the local oscillator signal derived from the same excitation source, $v_{LO}\left(t\right)=V_{LO}sin(\omega_{0}t)$, so that the mixer output $v_{m}\left(t\right)=v_{1}\left(t\right)v_{LO}(t)$ is:

$$v_{m}\left(t\right)=\frac{G_{1}\omega_{o}R_{s}V_{exc}V_{LO}}{2}\left[C_{0}+\Delta C\left(t\right)\right]\left[1+\cos\left(2\omega_{0}t\right)\right]$$

where *G*_1_ is the amplifier gain. The mixer employed (AD831, Analog Devices, Wilmington, MA, USA) can operate down to DC at its output, which allows for direct extraction of the low-frequency component proportional to the capacitance change. The mixer low-pass filter was set to have a cutoff frequency of 20 kHz. This bandwidth was chosen to preserve the dynamic content associated with mechanical slip events while efficiently rejecting the high-frequency components resulting from the mixing process and residual carrier leakage.

After low-pass filtering, the high-frequency component at $2\omega_{0}$ is removed, and the remaining baseband signal is

(19) $$v_{LP}\left(t\right)=\frac{G_{1}\omega_{o}R_{s}V_{exc}V_{LO}}{2}\left[C_{0}+\Delta C\left(t\right)\right]$$

The final signal conditioning stage provides additional amplification and DC component removal. Although represented as a single gain block in the schematic, this stage is implemented as two cascaded sections: a buffered single-pole high-pass filter, followed by the main voltage gain stage. The high-pass filter was designed with a cutoff frequency of 10 Hz, which suppresses slow drifts and offsets while retaining the relevant mechanical dynamics of the sensor response.

In fact, in Equation (19), the constant term proportional to C_0_ represents a DC offset, while the time-varying term proportional to ΔC(t) carries the information related to the mechanical excitation. The HP filter output is therefore:

(20) $$v_{HP}\left(t\right)=\frac{G_{1}\omega_{o}R_{s}V_{exc}V_{LO}}{2}\Delta C(t)$$

Finally, the signal is amplified by a second gain stage with gain *G*_2_ (overall gain of 40), scaling the demodulated signal to a level suitable for acquisition and further processing, so that:

(21) $$v_{s}\left(t\right)=K\Delta C\left(t\right),K=\frac{G_{1}G_{2}\omega_{o}R_{s}V_{exc}V_{LO}}{2}$$

Equation (21) shows that the output of the electronic front-end is directly proportional to the variation of the sensor capacitance and is independent of the nominal capacitance value.

As a result, the output of the front-end represents an amplified voltage signal directly proportional to the variation of the sensor capacitance and, consequently, to the mechanical stimuli applied to the device. Since static and parasitic contributions are effectively removed by the synchronous demodulation and filtering stages, the measured signal is dominated by dynamic effects related to slip-induced electrode motion. This electronic front-end was therefore employed for the dynamic characterization of the sensor and for the evaluation of its response both under controlled dynamic slip conditions and in real-world conditions.

## 5. Experimental Results

### 5.1. Dielectric Characterization

Prior to the construction of the prototype, the suitability of different fluidic dielectrics was systematically evaluated. A set of test formulations was prepared starting from the base fluids listed in Table 1, also including selected mixtures designed using a simple linear dynamic model to target a minimum mechanical cut-off frequency of at least 50 Hz, as computed according to the criteria reported in Table 2. Since pure glycerol, despite its very high relative permittivity, exhibited an excessively high effective viscosity, it was not used as a standalone dielectric but rather as an additive to low-viscosity silicone oils. In this way, the effective dielectric constant of the fluidic layer can be increased while maintaining the mechanical bandwidth within an acceptable range. The highest glycerol concentration experimentally investigated while still preserving a usable dynamic response was 33% (*W/W*).

For these tests, two planar electrodes were screen-printed on alumina substrates. Fixed spacers were bonded to one of the plates to define the gap thickness. Each dielectric formulation was then uniformly spread over the plate with spacers, and excess material was removed during the assembly of the second plate to ensure a reproducible and well-defined fluid layer. The electrodes had a diameter of 16 mm, while the inter-electrode distance was set to 300 µm using calibrated alumina spacers.

**Figure 6:**
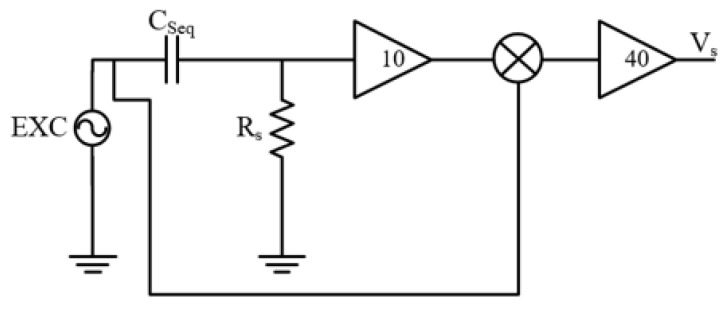
Block diagram of the developed front-end.

Capacitance (modeled using a simple resistance–capacitance equivalent circuit) and the impedance phase were subsequently measured over a representative frequency range from 10 kHz to 1 MHz (see Figure 6). This approach allowed for the simultaneous assessment of dielectric properties, losses, and formulation stability.

**Figure 7:**
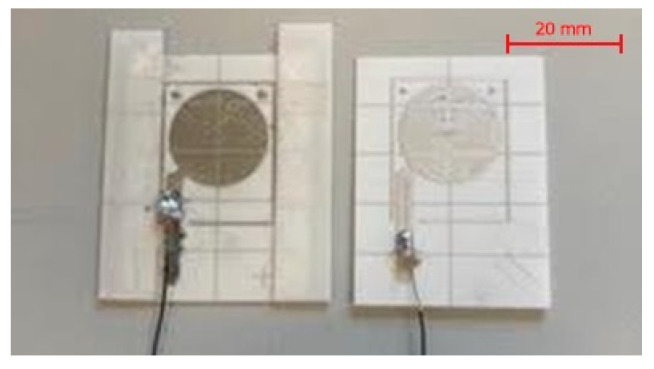
Test assembly for the evaluation of the fluid dielectric properties. Two plates were printed on alumina and spacers were fixed to the alumina base.

Experiments showed that the oil-based formulations became unstable over time, while the silicone pastes and glycerol- or white Vaseline-based formulations were generally stable (see Figure 7).

The results of the tests are reported in Table 2.

Furthermore, the results demonstrated that adding a small amount of glycerol to silicone increased the relative permittivity without affecting the dynamics, but the measured dielectric constant rose by only ~2.5× versus the predicted gain (Table 2). To improve the electrical sensitivity of the sensor while keeping viscosity within an acceptable range, the fluidic layer was modified by dispersing metal oxide nanoparticles characterized by low electrical conductivity and high dielectric constant. In particular, titanium dioxide (TiO_2_) nanoparticles were tested as dielectric fillers.

Titanium dioxide (TiO_2_) exhibits a very high relative permittivity, typically ε_r_ ≈ 80–100, and 30–50 for anatase, depending on crystal structure, particle size, and excitation frequency.

When dispersed in a low-permittivity medium such as silicone oil or petrolatum (ε_r_ ≈ 2.0–2.5), TiO_2_ nanoparticles can substantially increase the effective dielectric constant of the composite fluid while preserving reasonable flow characteristics, provided that the loading concentration remains moderate (typically below 1 wt%).

At higher concentrations, particle agglomeration and increased hydrodynamic drag lead to a marked rise in apparent viscosity, which in turn increases the sensor time constant τ=ηA/(kd).

Note that nanoparticle loading tends to increase the effective viscosity of the dielectric medium and, consequently, affects the mechanical time constant τ of the sensing structure. The use of TiO_2_-enhanced lubricants and suspensions is well-documented in the literature. For instance, Wei et al. demonstrated that TiO_2_-based nanolubricants exhibit improved rheological and tribological performance, including viscosity modulation and enhanced load-carrying capability [19]. Similarly, Ratoi et al. investigated the influence of TiO_2_ nanoparticles on the rheological and frictional behavior of lubricating media, highlighting their potential for tailoring dynamic response characteristics [20]. These findings confirm the feasibility of using TiO_2_ nanoparticle suspensions as dielectric–viscous hybrids, suitable for capacitive tactile sensing applications.

Therefore, the concentration and dispersion stability were optimized to balance dielectric enhancement and mechanical response.

Tests were performed using the tested viscous fluids as carriers, formulated with different % (*W/W*) of TiO_2_; some results are reported in Table 3.

### 5.2. Sensor Characterization

#### 5.2.1. Calibration Under Controlled Tangential Displacement

A set of sensors realized with the fluids selected through the previous characterization were tested. In detail, sensors with silicone oil, a mixture of silicone oil (66%) and glycerol (33%), and finally silicone oil (95% *W/W*) with TiO_2_ nanoparticles (5% *W/W*) were machined and tested.

The developed sensors were first characterized on the test bench presented in Section 4 by imposing a known tangential displacement between electrodes using a linear piezo actuator. The electrodes were bonded to rigid 3D-printed plates (see Figure 5) to ensure defined kinematics and minimize parasitic deformation.

**Figure 8:**
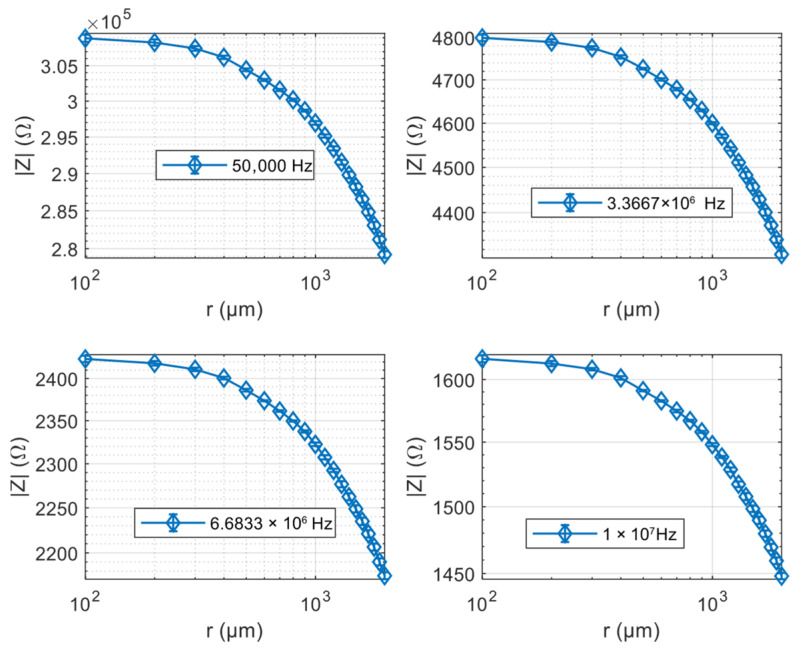
Impedance characterization of the proposed soft capacitive slip sensor, dielectric silicone oil, and measured impedance magnitude as a function of the relative displacement between the two electrodes, acquired at different excitation frequencies.

At each commanded displacement step (100 μm), the impedance analyzer (Wayne Kerr 6500B) executes a frequency sweep over the characterization range and records both the magnitude and phase of the impedance. All of the tested sensors, with different fluid dielectric, behave as quasi-pure capacitances, and above 50 kHz, the impedance module has values that allow for easy measurements, as shown in Figure 8, where representative data are reported.

Under static conditions, the devices have typical capacitance values between 8 and 15 pF, depending on the dielectric formulation. From impedance measurements performed at frequencies above 100 kHz, the equivalent capacitance was calculated assuming a purely capacitive impedance, yielding results with a dispersion well below 1%.

**Figure 9:**
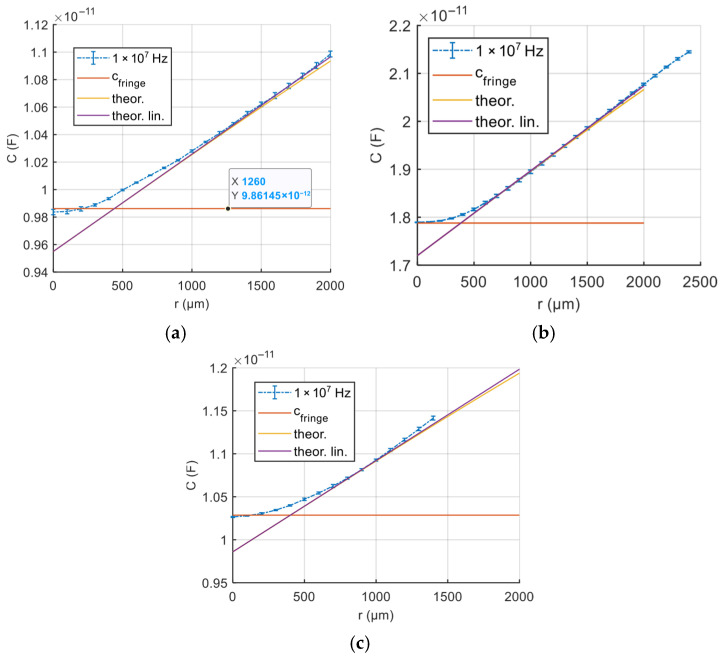
Static calibration of the capacitive slip sensor under controlled shear displacement for different dielectric formulations. (**a**) Silicone oil, (**b**) silicone–glycerol mixture, and (**c**) silicone–TiO_2_ composite. The capacitance is plotted as a function of the relative electrode displacement, *r*, measured at an excitation frequency of 10 MHz. For each formulation, 20 calibration cycles were repeated. Error bars report mean and standard deviation. The solid line corresponds to the analytical model, the dashed line to the quasi-linear approximation, and the constant offset highlights the contribution of fringe capacitance. The reference displacement is the true position provided by the linear actuator.

Figure 9 shows the measured capacitance variation (at 1 MHz) compared to the relative displacement for sensors with different dielectrics. Experimental data are in line with the analytical model introduced in Section 2. In particular, the experimental data followed the predicted quasi-linear trend associated with the variation of the electrode overlap area, except for the small displacement range. In fact, as expected, for small displacements, deviations from the quasi-linear model were observed. These discrepancies can be attributed to a combination of electrical and mechanical effects that are not explicitly captured by the simplified analytical formulation.

From an electrical standpoint, fringe-field contributions become non-negligible when the relative displacement is small, as discussed in Section 2. Conversely, from a mechanical perspective, the onset of motion is influenced by static friction and micro-adhesion phenomena. At small displacements, the relative motion between the electrodes may be influenced by stick–slip behavior at the fluid–solid interfaces. These effects introduce a nonlinear compliance that reduces the effective sensitivity in the initial displacement range.

Despite these second-order effects, the overall response remained well-described by the proposed model with an expected resolution of about 200 μm and over the operating displacement range relevant for slip detection, confirming its suitability for calibration and practical use.

#### 5.2.2. Effect of Normal Load and Film Thickness

**Figure 10:**
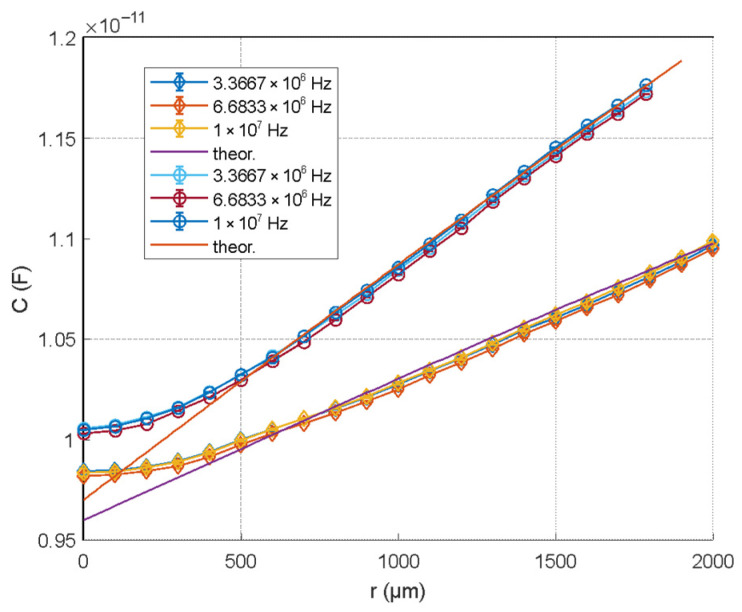
Effect of normal load on the static capacitance–displacement response of the sensor. Capacitance as a function of relative electrode displacement measured at three excitation frequencies (3.37 MHz, 6.68 MHz, and 10 MHz) under two different normal loads: approximately 5 N (corresponding to a 0.5 kg mass) and approximately 25 N (corresponding to a 2.5 kg mass). Solid lines represent analytical model predictions, while markers indicate experimental data.

To investigate the influence of normal pressure on the sensor response, a series of measurements were performed by applying different normal loads (5 N and 25 N) while repeating the same tangential displacement protocol. In these tests, the sensor was not bonded to the driving plates; instead, it was dragged by the plates through frictional contact, allowing the relative motion to be transmitted without adhesive constraints. The results, summarized in Figure 10, show that the capacitance–displacement relationship is weakly affected by the applied pressure.

This behavior is mainly attributed to variations in the effective thickness of the viscous dielectric layer, which was estimated to decrease from approximately 500 µm to 350 µm over the explored load range. As expected from the capacitive formulation, a reduced gap led to an increase in absolute capacitance and to a moderate change in sensitivity. Despite these variations, the overall shape of the response remained consistent, indicating that the sensor behavior is robust against moderate changes in normal load, a relevant condition for practical manipulation scenarios.

Although the capacitance–displacement slope showed only a weak dependence on normal load, time-varying grasp forces in closed-loop control could introduce a moderate gain modulation in the shear-sensitive response due to pressure-induced changes in the viscous layer thickness. This residual dependence can be compensated structurally by exploiting the stacked architecture of the sensor. In particular, an additional full-area electrode can be arranged facing the shield plate, separated by a compliant solid dielectric. Alternatively, the capacitance formed between the outer solid electrode and the inner electrode can be exploited for this purpose, although in this configuration, the electrostatic shielding effectiveness is reduced. The capacitance of this auxiliary channel is predominantly sensitive to normal compression and only marginally affected by tangential displacement. By monitoring this second capacitance, the instantaneous normal-load condition can be estimated and used to normalize or adaptively correct the slope of the shear-sensitive channel. This dual-channel configuration enables the compensation of pressure-induced sensitivity variations and enhances robustness in closed-loop grasp control scenarios involving time-varying normal forces

#### 5.2.3. Durability and Repeatability Under Cyclic Loading

Durability tests were conducted to assess the stability of the sensor under repeated tangential loading. Fast cyclic displacement tests were performed for up to 500 cycles. In this configuration, the sensor was not rigidly bonded to the actuation plates; instead, the encapsulated sensor was placed between two rigid surfaces and loaded with a normal force of approximately 10 N. This setup more closely resembles real operating conditions, where the deformation of the surrounding compliant structure contributes to the overall displacement.

**Figure 11:**
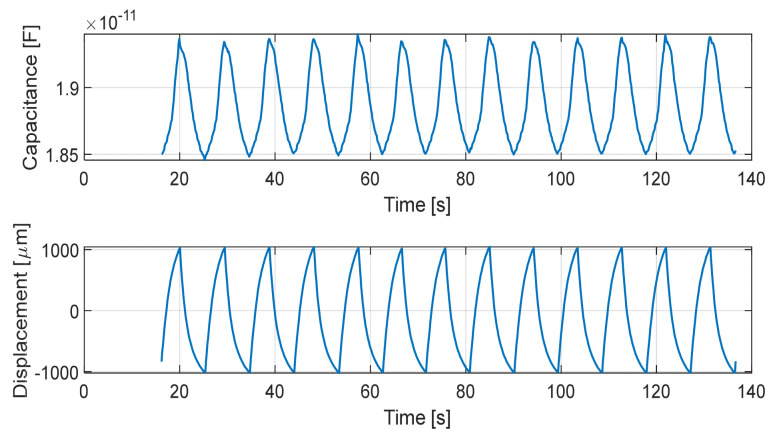
Measured capacitance response of the sensor during a 500-cycle durability test under repeated tangential loading. The first 13 cycles are reported for clarity, being representative of the behavior observed over all 500 cycles.

Due to this compliant coupling, a difference between the imposed actuator displacement and the effective electrode displacement was observed, caused by deformation of the intermediate layers. An example of the capacitance response over 13 representative cycles is shown in Figure 11, demonstrating good repeatability and the absence of significant drift or degradation.

The stability of the device was quantified by computing the peak-to-peak capacitance span at different intervals. We observed an average capacity span of 0.87 pF in the first 10 trials and 0.89 pF over the first 100 trials. Toward the end of the experiment, the average span was 0.89 pF for the last 100 trials and 0.89 pF for the final 10 trials.

The total variation throughout the 500-cycle test remained below 5%, demonstrating that the sensor provides a consistent response without significant monotonic drift or signal degradation. This confirms that the silicone encapsulation effectively maintains the mechanical integrity of the fluid dielectric layer during prolonged operation.

The encapsulation of the sensor introduces additional compliance and damping, increasing the effective mechanical time constant of the structure. However, slip and incipient slip events occur at the external silicone–object interface and are associated with localized and rapid releases of shear stress. These stress transients are mechanically transmitted through the silicone layer and coupled to the internal viscous dielectric, producing measurable capacitance variations that are not governed by the quasi-static response of the encapsulated structure, as will be shown in the next subsection

#### 5.2.4. Tests on a Robotic Finger During Grasping and Slip

**Figure 12:**
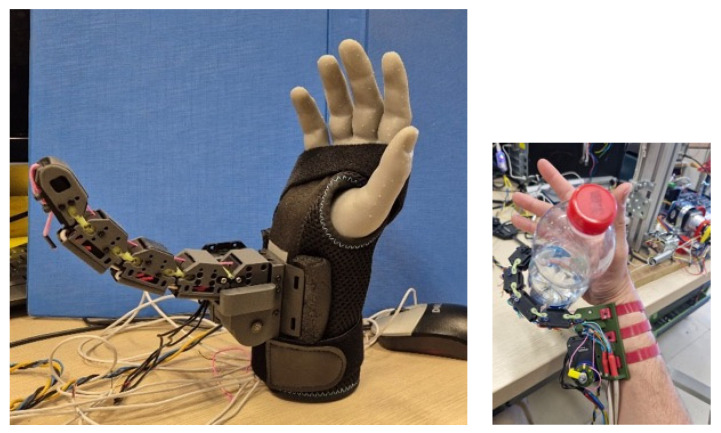
Robotic finger used for the tests.

To evaluate the sensor in a real scenario, it was integrated into the robotic finger described in [21,22,23] (Figure 12) and tested in grasping and slip experiments

The experiment consisted of grasping an object with the robotic finger; in particular, the tests were conducted using a water bottle. The finger was brought into contact with the bottle and loaded to a normal force of approximately 5 N, after which grasping and controlled slip were performed while the sensor capacitance was recorded with a laboratory impedance analyzer at a sampling interval of ~100 ms.

**Figure 13:**
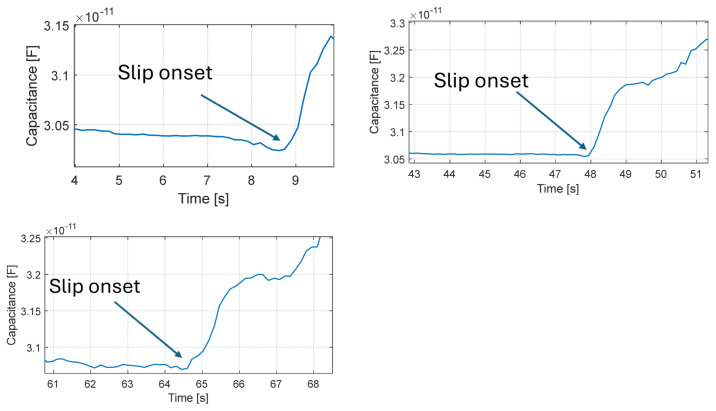
Slip detection experiments performed using the proposed capacitive sensor integrated into a robotic finger during grasping of a plastic bottle. The plots show the time evolution of the measured capacitance during repeated grasping trials. Capacitance was measured at 10 MHz using the impedance analyzer.

As can be seen in Figure 13, slip events were mainly observable in the low-frequency content of the signal, appearing as variations associated with the relative displacement of the electrodes. In all trials, the onset of slip corresponded to a sudden capacitance change, associated with relative tangential motion between the encapsulated electrodes induced by object slippage. With this setup, high-frequency components were not observable due to the low-pass behavior of the measurement system used for capacitance read-out.

#### 5.2.5. Slip Experiments on Different Surfaces: Dynamic Response

To capture higher-frequency phenomena associated with slip, additional experiments were performed using the dedicated custom electronic readout described in Section 3.

In this experiment, the sensor was repeatedly slid over surfaces with different textures (smooth plastic, rough plastic and metal). Under these conditions, the measured signal exhibited pronounced high-frequency components arising from surface-induced vibrations and stick–slip interactions. These vibrations were amplified by the capacitive transduction mechanism and varied systematically with the surface properties, corroborating the sensor’s capability to capture dynamic signatures associated with slip and surface contact.

The dynamic friction coefficients associated with the sliding of silicone rubber against a smooth plastic surface are typically in the range of approximately 0.5–1.2, increasing to about 0.8–1.8 for highly rough plastic surfaces, and ranging between 0.7 and 1.5 for metal contacts, under dry and unlubricated conditions, in agreement with tribological data reported for silicone elastomers. These values indicate that the different surfaces involved in the experiments are characterized by friction coefficients of the same order of magnitude while still exhibiting significant variations due to surface texture and material properties.

In the experiments reported here, the sensor was pressed against the test surfaces with a normal force of approximately 5 N.

**Figure 14:**
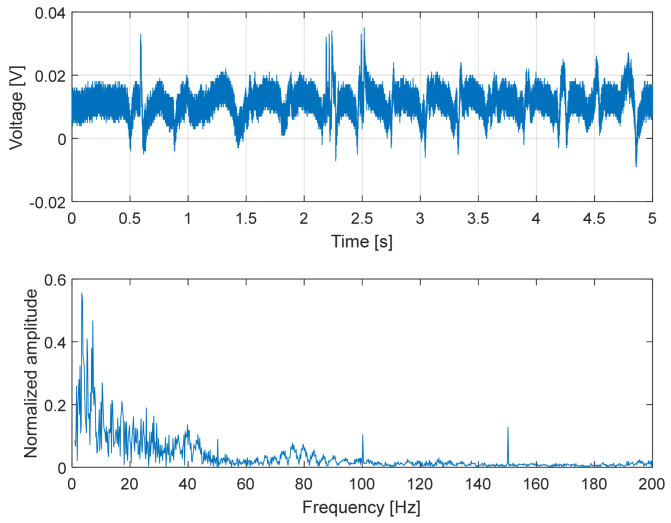
Dynamic response of the capacitive slip sensor during sliding on a smooth plastic surface. (**Top**): time evolution of the demodulated capacitance signal acquired using the custom high-frequency readout, highlighting slip-induced fluctuations during relative motion. (**Bottom**): corresponding amplitude spectrum, showing limited high-frequency content and relatively smooth vibration signatures, consistent with the low surface roughness and reduced stick–slip activity of the contacted material.

**Figure 15:**
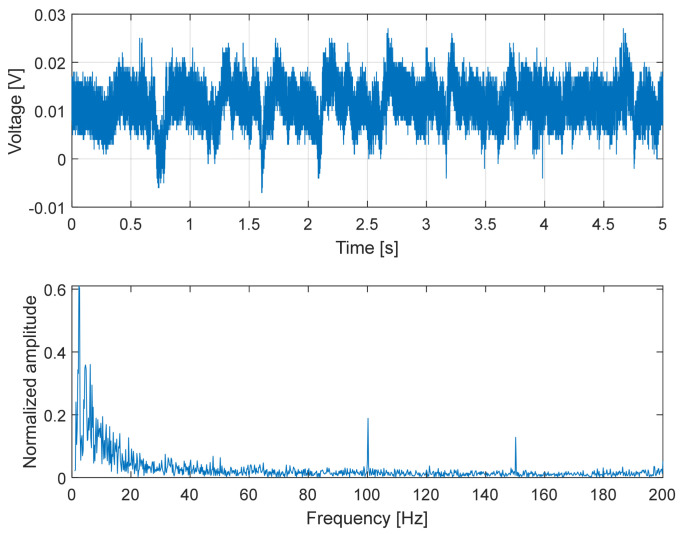
Dynamic response of the capacitive slip sensor during sliding on a rough plastic surface. (**Top**): time-domain capacitance signal exhibiting increased amplitude modulation and irregular fluctuations associated with enhanced stick–slip phenomena. (**Bottom**): frequency-domain representation, revealing a broader spectral content and higher energy at mid-to-high frequencies compared to smooth plastic, due to surface texture-induced micro-vibrations.

**Figure 16:**
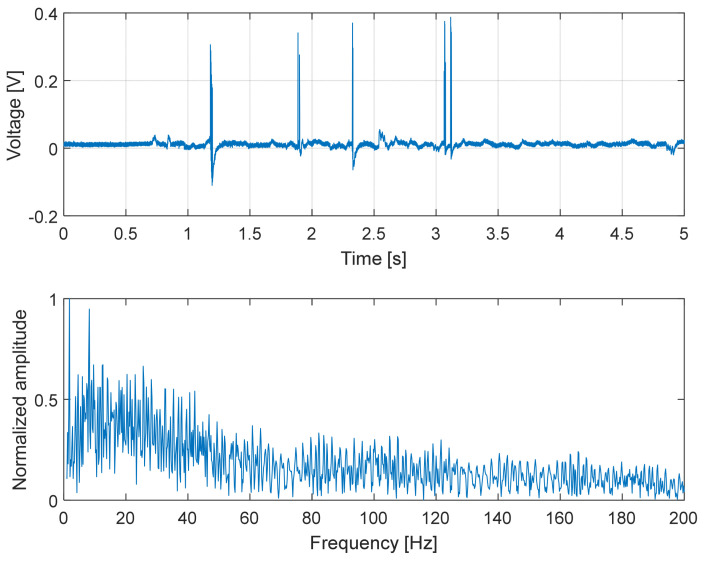
Dynamic response of the capacitive slip sensor during sliding on a metal surface. (**Top**): time-domain capacitance signal characterized by sharp transient peaks corresponding to abrupt slip events and micro-detachments at the contact interface. (**Bottom**): amplitude spectrum showing pronounced high-frequency components, indicative of strong frictional interactions and reduced damping at the metal–sensor interface.

Overall, the results presented in Figure 14, Figure 15 and Figure 16 demonstrate that the proposed capacitive slip sensor is capable of discriminating different contact conditions through their dynamic signatures. While the absolute signal amplitude depends on the applied load and sliding conditions, the observed differences in temporal structure and spectral content highlight the sensitivity of the sensor to surface texture and material properties, supporting its use as a qualitative indicator of contact dynamics rather than as a binary slip detector.

A comparison between the modeled mechanical bandwidth and the experimentally observed spectral content further supports the validity of the simplified dynamic model reported in Section 2.2. For the selected configuration (silicone oil 100 cSt), Table 1 predicted an intrinsic mechanical cut-off frequency of approximately 160 Hz, derived from the first-order over-damped model. This value represents the ideal bandwidth of the shear-sensing structure without considering additional external compliance.

In the final prototype, however, the sensing stack is fully encapsulated in silicone rubber, which introduces additional tangential compliance and damping. As a result, the effective mechanical bandwidth of the complete device is expected to remain of the same order of magnitude but lower than the modeled value.

This behavior is consistent with the experimental results reported in Figure 14, Figure 15 and Figure 16. The dominant spectral components during sliding lay in the range of a few tens of hertz. In particular, Figure 14 shows significant components around ~80 Hz, while the amplitude spectrum in Figure 16 exhibited a roll-off compatible with an effective −3 dB bandwidth in the order of 60–70 Hz. The agreement in order of magnitude between the predicted and measured bandwidth indicates that the simplified over-damped model provides a reliable design guideline for selecting the dielectric viscosity and geometry to target the frequency range relevant for slip dynamics (10–100 Hz), while acknowledging that encapsulation effects slightly reduce the effective bandwidth of the final device.

## 6. Conclusions and Future Work

This work presents the modeling, design, fabrication, and experimental validation of a soft capacitive tactile sensor specifically conceived to capture shear-driven contact dynamics in robotic manipulation. The proposed sensing principle differs from conventional pressure-oriented capacitive architectures by exploiting a viscous dielectric interlayer that enables controlled in-plane relative motion between flexible electrodes, converting tangential micro-displacements into measurable capacitance variations.

A simplified analytical model was introduced to describe the relationship between shear-induced relative displacement and the resulting capacitive response. Despite its intentional simplicity, the model proved effective in guiding geometry selection and dielectric formulation, and was experimentally validated under controlled tangential displacement. The concentric-ring electrode geometry demonstrated an approximately isotropic response with respect to slip direction, while the stacked and shielded configuration significantly reduced sensitivity to normal compression and parasitic effects.

Experimental characterization confirmed a quasi-linear response over the displacement range relevant to slip, with small-displacement deviations attributed to fringe fields, static friction, and micro-adhesion. Tests under varying normal loads showed only a weak sensitivity to pressure-induced dielectric thinning, indicating robustness to moderate grasp-force changes. Cyclic durability tests further demonstrated stable, repeatable performance with no appreciable degradation over hundreds of cycles.

A key aspect of the proposed approach is the intentional separation between static and dynamic information. Static or quasi-static capacitance levels are strongly influenced by object geometry and equilibrium contact conditions and are therefore poorly suited for absolute slip estimation. In contrast, slip events manifest as dynamic, non-linear transients and surface-dependent vibrations that remain clearly observable even when the overall mechanical time constant of the encapsulated structure is relatively large. This behavior was confirmed both in the controlled test-bench experiments and during proof-of-concept integration into a robotic finger.

Slip experiments performed on different surfaces—smooth plastic, highly rough plastic, and metal—highlighted that the sensor is sensitive not only to the occurrence of slip, but also to the nature of the contact. Distinct temporal and spectral signatures were observed depending on surface texture and material, reflecting differences in frictional interaction and stick–slip dynamics. These results indicate that the proposed sensor can provide qualitative information about contact conditions and surface-dependent dynamics, rather than acting as a binary slip switch.

From an instrumentation perspective, the dedicated high-frequency excitation and synchronous demodulation readout proved effective in isolating dynamic capacitance variations while rejecting large static offsets and parasitic contributions. This architecture enables compact implementation and is well-suited for integration in soft robotic platforms.

Overall, the results demonstrate that shear-sensitive capacitive sensing with a fluid dielectric interlayer represents a viable and low-complexity approach for monitoring contact dynamics in robotic manipulation. Rather than aiming at precise friction estimation or absolute slip quantification, the proposed sensor is best interpreted as a robust indicator of tangential interaction dynamics, capable of supporting grasp monitoring and adaptive control strategies.

It is worth noticing that while the proof-of-concept experiments confirmed the sensor’s ability to monitor contact dynamics in a real-world robotic integration, some limitations must be acknowledged. The current study focused on a single object (a plastic water bottle) and a specific grasping posture with a controlled normal force. Consequently, the influence of varying object shapes, weights, and material properties on the sensor’s absolute response was not exhaustively characterized. Furthermore, the experiments were conducted under open-loop conditions without exploring the impact of different grasping strategies, such as active force or position control. These factors are critical for a universal slip-sensing solution. Future research will be dedicated to evaluating the sensor across a broader library of objects and integrating its dynamic output into closed-loop control schemes to enable real-time grasp adaptation and slip mitigation. Moreover, we will focus on extending the dynamic characterization to a broader range of materials and loading conditions, refining the rheological modeling of the dielectric layer to account for non-Newtonian effects, and integrating the sensor output into closed-loop control schemes for real-time grasp adaptation and slip mitigation.

## Figures and Tables

**Table 1:** Expected dynamic features of the proposed sensor.

Fluid	Dynamic Viscosity η [Pa·s]	Damping b = ηA/d [N·s/m]	Time Constant τ = b/k [s]	Cut-Off Freq. fc = 1/(2π τ) [Hz]	Relative Permittivity ε_r_
Isopropyl alcohol (IPA)	0.002	2.0	2 × 10^−5^	8 × 10^3^	20–25
Silicone oil 100 cSt	0.1	100	1 × 10^−3^	160	2.5
Silicone oil 1000 cSt	1.0	1 × 10^3^	1 × 10^−2^	16	2.5
Glycerol (pure)	1.2	1.2 × 10^3^	1.2 × 10^−2^	13	42
Vaseline 100%	30–80	3 × 10^4^–8 × 10^4^	0.3–0.8	0.2–0.5	1.8–2.0
Automotive lubricating oil (10 W–40 grade)	0.2–0.4	200–400	2–4 × 10^−2^	40–80	2.1–2.3

**Table 2:** Dielectric characterization results.

Fluid/Mixture	Theoretical Capacitance Value (pF)	Measured Capacitance (pF)	Phase (°)	Frequency (kHz)	Stability
Air	4.0	5.8	−89.8	10	Stable
Isopropyl alcohol (IPA)	80	470.0 82.8	−25 −88.6	10 1000	Very poor dielectric, problems with electrical polarization in the presence of DC electric voltage
Silicon oil 100 cSt	10	14.3	−89.9	10	Stable
Glycerol 100%	168	199.0 162.0	−66.2 −89.9	10 1000	Poor dielectric, problems with electrical polarization in the presence of DC electric voltage
Automotive lubricating oil (10 W–40 grade)	10	11.4	−88.4	10	Not compatible with the process of encapsulation in silicon
Silicon oil 100 cSt 66.6%-Glycerol 33.3% (*W/W*)	114	25.2	−86.45	10	Stable

**Table 3:** Dielectric characterization results, silicone oil and paste.

Fluid/Mixture	Theoretical Capacitance Value (pF)	Measured Capacitance (pF)	Phase (°)	Frequency (kHz)	Stability
Silicone oil 100 cSt 95%–TiO_2_ 5% (*W/W*)	10.4	15	−89.6	10	stable
Silicone oil 100 cSt 50%–TiO_2_ 50% (*W/W*)	16.4	24.7	−89.6	10	stable

## Data Availability

All the data are embedded in the article.

## References

[1] Mason M.T., Salisbury J.K. (1985). Robot Hands and the Mechanics of Manipulation.

[2] Howe R.D., Cutkosky M.R. (1996). Practical force-motion models for sliding manipulation. Int. J. Robot. Res..

[3] Cutkosky M.R., Wright P.K. (1987). Skin materials for robotic fingers. Proceedings of the IEEE International Conference on Robotics and Automation.

[4] Hogan N. (1985). Impedance control: An approach to manipulation. J. Dyn. Syst. Meas. Control.

[5] Prattichizzo D., Trinkle J.C. (2016). Grasping. Springer Handbook of Robotics.

[6] Romano J.M., Hsiao K., Niemeyer G., Chitta S., Kuchenbecker K.J. (2011). Human-inspired robotic grasp control with tactile sensing. IEEE Trans. Robot..

[7] Dahiya R.S., Metta G., Valle M., Sandini G. (2010). Tactile sensing—From humans to humanoids. IEEE Trans. Robot..

[8] Romeo R.A., Zollo L. (2020). Methods and sensors for slip detection in robotics: A survey. IEEE Access.

[9] Johansson R.S., Flanagan J.R. (2009). Coding and use of tactile signals from the fingertips in object manipulation tasks. Nat. Rev. Neurosci..

[10] Yousef H., Boukallel M., Althoefer K. (2011). Tactile sensing for dexterous in-hand manipulation in robotics—A review. Sens. Actuators A Phys..

[11] Yuan W., Dong S., Adelson E.H. (2017). GelSight: High-resolution robot tactile sensors for estimating geometry and force. Sensors.

[12] Li R., Adelson E.H. (2015). Improved GelSight tactile sensor for measuring geometry and slip. Proceedings of the IEEE/RSJ International Conference on Intelligent Robots and Systems.

[13] Donlon E., Dong S., Liu M., Li J., Adelson E.H., Rodriguez A. GelSlim: A high-resolution, compact, robust, and calibrated tactile-sensing finger. Proceedings of the 2018 IEEE/RSJ International Conference on Intelligent Robots and Systems (IROS).

[14] Agarwal A., Li R., Adelson E.H. (2025). A modularized design approach for the GelSight family of vision-based tactile sensors. IEEE Robot. Autom. Lett..

[15] Li S., Wang Z., Wu C., Li X., Luo S., Fang B., Sun F., Zhang X.P., Ding W. (2017). When vision meets touch: A contemporary review for visuotactile sensors from the signal processing perspective. IEEE Sens. J..

[16] Liu C., Huh T.M., Chen S.X., Lu L., Kopsaftopoulos F., Cutkosky M.R., Chang F.K. (2022). Design of active sensing smart skin for incipient slip detection in robotics applications. IEEE/ASME Trans. Mechatron..

[17] Jamali N., Sammut C. (2011). Majority voting: Material classification by tactile sensing using surface texture. IEEE Trans. Robot..

[18] Melchiorri C. (2002). Tactile sensing for robotic manipulation. Ramsete: Articulated and Mobile Robotics for Services and Technologies.

[19] Birleanu C., Pustan M., Cioaza M., Molea A., Popa F., Contiu G. (2022). Effect of TiO_2_ nanoparticles on the tribological properties of lubricating oil: An experimental investigation. Sci. Rep..

[20] Del Río J.M.L., Mariño F., López E.R., Gonçalves D.E., Seabra J.H., Fernández J. (2023). Tribological enhancement of potential electric vehicle lubricants using coated TiO_2_ nanoparticles as additives. J. Mol. Liq..

[21] Landi E., Baldi T.L., Papenbrock J., Prattichizzo D., Fort A., Riccio M. (2025). Sensor-Equipped Joint Design for Accurate Trajectory Tracking in Soft Robots. Proceedings of the 2025 IEEE International Workshop on Metrology for Industry 4.0 & IoT (MetroInd4.0 & IoT).

[22] Landi E., Baldi T.L., Papenbrock J., Facello A., Prato A., Schiavi A., Fort A. (2025). Sensorizing Flexible Joints for Soft Robots: A Feasibility Study. Proceedings of the 2025 IEEE International Instrumentation and Measurement Technology Conference (I2MTC).

[23] Landi E., Baldi T.L., Papenbrock J., Facello A., Prato A., Schiavi A., Fort A. (2025). Sensorization of Soft Robot Joints for Accurate and Durable Bending Measurement. IEEE Sens. J..

